# Self-Assembly of Nanodiamonds and Plasmonic Nanoparticles for Nanoscopy

**DOI:** 10.3390/bios12030148

**Published:** 2022-02-28

**Authors:** Lukas Schmidheini, Raphael F. Tiefenauer, Volker Gatterdam, Andreas Frutiger, Takumi Sannomiya, Morteza Aramesh

**Affiliations:** 1 Laboratory of Biosensors and Bioelectronics, Institute for Biomedical Engineering, ETH Zürich, 8092 Zürich, Switzerland; lukas.schmidheini@biol.ethz.ch (L.S.); raphael.tiefenauer@gmail.com (R.F.T.); v.gatterdam@lino-biotech.com (V.G.); frutiger@biomed.ee.ethz.ch (A.F.); 2 Department of Materials Science and Engineering, School of Materials and Chemical Technology, Tokyo Institute of Technology, Yokohama 226-8503, Japan; sannomiya.t.aa@m.titech.ac.jp; 3 Department of Materials Science and Engineering, Division of Biomedical Engineering, Uppsala University, 751 21 Uppsala, Sweden

**Keywords:** blinking nanodiamonds, gold nanoparticles, plasmonic coupling, multiphoton excitation, nanoscopy

## Abstract

Nanodiamonds have emerged as promising agents for sensing and imaging due to their exceptional photostability and sensitivity to the local nanoscale environment. Here, we introduce a hybrid system composed of a nanodiamond containing nitrogen-vacancy center that is paired to a gold nanoparticle via DNA hybridization. Using multiphoton optical studies, we demonstrate that the harmonic mode emission generated in gold nanoparticles induces a coupled fluorescence emission in nanodiamonds. We show that the flickering of harmonic emission in gold nanoparticles directly influences the nanodiamonds’ emissions, resulting in stochastic blinking. By utilizing the stochastic emission fluctuations, we present a proof-of-principle experiment to demonstrate the potential application of the hybrid system for super-resolution microscopy. The introduced system may find applications in intracellular biosensing and bioimaging due to the DNA-based coupling mechanism and also the attractive characteristics of harmonic generation, such as low power, low background and tissue transparency.

## 1. Introduction

Nanodiamonds are attractive biomarkers in sensing applications because of their strong and stable fluorescence emission, robust structure and excellent biocompatibility [1,2,3]. Fluorescence emission in nanodiamonds is due to naturally occurring or implanted crystal defects, such as nitrogen vacancies (NV^0^ and NV-centers) [4,5]. The optical transitions in NV centers are sensitive to electromagnetic fields, temperature, pH and the nanoscale environment, enabling the localized sensing of small changes in complex environments [6,7,8]. For example, a nanodiamond can be integrated with an atomic force microscope (AFM) to scan across a cell membrane or other surfaces, via measurement of the quantum decoherence of the NV probe [9,10]. Particularly, NV centers in nanodiamonds have been utilized for the imaging of cells, organisms and animals [11,12,13,14]. More recently, with the rise of super-resolution microscopy (SRM) [15,16], NV centers have attracted attention as potential probes for SRM [17].

In this work, nanodiamond–gold nanoparticle (GNP) conjugates were formed via self-assembling DNA hybridization and the photoemission of the conjugates was investigated by multiphoton laser excitation. We demonstrated that the NV centers in nanodiamonds can be excited via coupling with the GNPs’ second harmonic emission. The flickering of the harmonic surface plasmon modes in GNPs induced blinking in the nanodiamond emission. We discussed the potential of the introduced system for super-resolution microscopy.

## 2. Functionalization and Hybridization Assay

**Figure 1:**
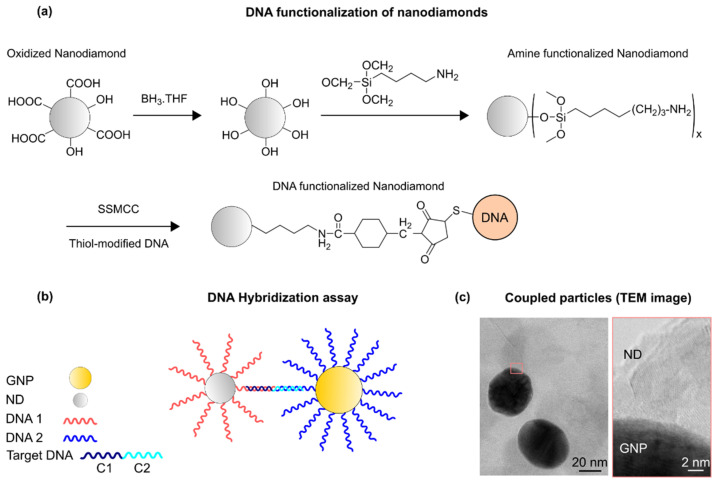
(**a**) DNA functionalization of nanodiamonds. (**b**) Principles of DNA-induced nanoassembly. DNA-functionalized nanoparticles form conjugates via hybridization by a target DNA that contains sequences complementary to both GNP and nanodiamond tags. Nanodiamonds and GNPs are functionalized with DNA1 and DNA2, respectively. The target DNA is composed of C1 and C2, complementary sequences to DNA1 and DNA2, respectively. (**c**) A transmission electron microscope (TEM) image of a coupled GNP–nanodiamond structure.

The principle of the DNA-controlled assembly of a GNP-nanodiamond system is shown in Figure 1. The concept builds on our previous study, where a target DNA was used to couple two differently-tagged GNPs [18,19]. The target DNA is composed of two parts, in which half of the sequence is complementary to the GNP tags and the other half to the nanodiamond tags. Upon DNA hybridization, the conjugated GNP–nanodiamond pairs form in the solution. The distance between the particles and the ratio of the coupled particles can be controlled by length and concentration of the target DNA, respectively [18,20]. Surface functionalization was performed on each component separately prior to the DNA assembly assay. Nanodiamond functionalization was performed using previously established protocols. Briefly, the surface of the nanodiamonds was oxidized by annealing the particles at 600 °C in air [21], and then a two-step chemistry was performed to attach DNA to the particles. In the first step, the surface of the nanodiamonds was functionalized with amine (-NH_2_) groups [22,23]. Subsequently, an SSMCC crosslinker, sulfosuccinimidyl-4-(N-maleimidomethyl)cyclohexane-1-carboxylate), was used to react with the thiol-modified DNAs [24,25]. The average size of the particles was determined using dynamic light scattering (DLS). The DNA assembly assay was then performed in designed microwells, whose details are described in the Supplementary Materials. For the optical analysis, the conjugated assemblies were coated on glass coverslips.

## 3. Optical Properties of Nanodiamonds and GNPs

**Figure 2:**
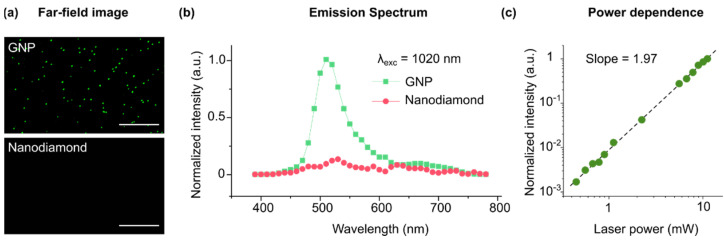
Multiphoton emission from single GNPs and nanodiamonds. (**a**) Far-field optical images obtained by multiphoton excitation of GNPs and nanodiamonds at λ = 1020 nm and ~1 mW excitation power. No detectable signal was observed in the nanodiamonds (at λ = 1020 nm and ~1 mW). Scale bars, 10 µm. (**b**) The corresponding average emission spectrum of the GNP and nanodiamonds. GNP emission was centered at 520 nm. (**c**) The emission intensity in GNP increases with excitation power almost quadratically (slope = 1.97 in the log scale).

The optical properties of GNPs are intimately linked to localized surface plasmon (LSP) resonances, which stem from the collective oscillations of the conduction electrons of metals upon excitation by an oscillating electromagnetic field [26,27]. LSP resonances can locally couple with the electronic states of other materials [28,29,30,31,32,33,34,35]. The resonance band of the LSPs in GNP is size-dependent, and it lies in the visible spectrum for particles smaller than 100 nm [36]. GNPs of 50 nm size were chosen for this study because their LSP resonance is centered at 520 nm, matching the excitation wavelength of the NV centers (see Supplementary Materials). We determined whether the near-field LSP resonance in 50 nm GNP was sufficient to excite the NV center in the coupled nanodiamond particles. Our multiple multipole program modeling (MMP [37]) suggested that the surface plasmons in GNP produce a strong electromagnetic field near the particle which spatially covers the coupled nanodiamond particle (see Supplementary Materials). The surface field strength directly corresponds to two-photon generation probability (*E*^2^). Since white light or linear laser illuminations simultaneously excite LSPs in GNPs and NV centers in nanodiamonds, it was not possible to investigate the coupling using this method. Therefore, it was necessary to separate the excitation sources for GNPs and NV centers in the experimental setup. For this purpose, we investigated the suitability of multiphoton microscopy for the excitation of harmonic surface plasmon modes in GNPs. In agreement with previous reports, a wavelength-dependent emission was observed in the spectrum of individual GNPs under multiphoton illumination [38,39,40,41,42,43,44]. At λ = 1020 nm excitation, GNPs exhibited a dominating far-field emission centered at 520 nm (Figure 2). The emission power was quadratically proportional to the excitation power (with a slope of 1.97 in the logarithmic scale), suggesting that the emission was due to the plasmonic excitation of the particles via two-photon absorption. On the other hand, no detectable emission was observed in individual nanodiamonds at the investigated conditions (illumination power up to 1.2 mW and excitation wavelengths of 800–1300 nm. Supplementary Materials). It is noteworthy to mention that the shape of GNPs may influence the harmonic emission of the particles due to their anisotropic effects. The GNPs used in this study might deviate from a perfectly spherical shape (DLS data in Supplementary Materials). Depending on its shape, each individual particle may exhibit different optical properties, however, the signal obtained from an ensemble of GNPs should reflect the average optical properties of all particles.

## 4. Emission Coupling between GNP–Nanodiamond Pairs

**Figure 3:**
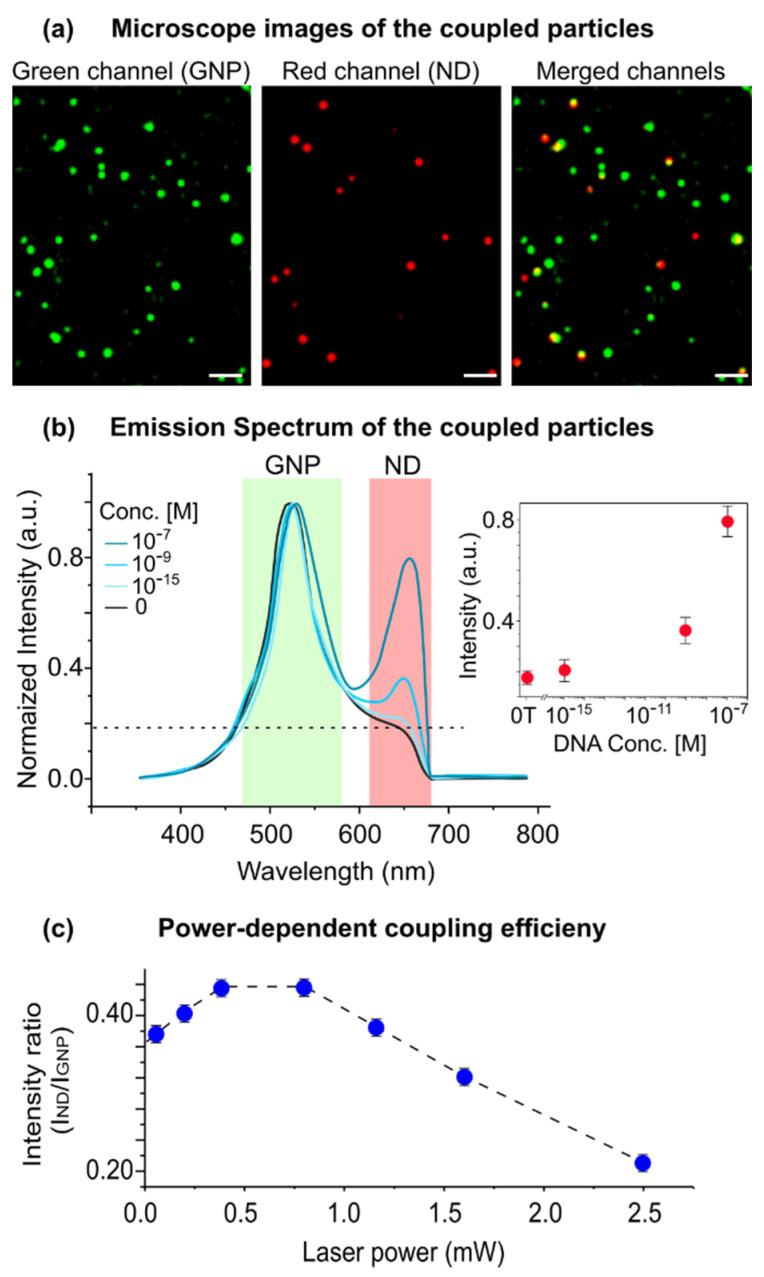
Optical properties of the coupled GNP–ND system. (**a**) Far-field optical images of the coupled system under multiphoton excitation (λ_excitation_ = 1020 nm laser power ~1 mW). Green and red channels correspond to emission in the 480–580 nm (GNPs) and 620–680 nm (nanodiamonds) ranges, respectively. A total of 95% of the emission centers in the red channels are colocalized with the emission centers in the green channel. Scale bars, 4 µm. (**b**) The spectrum of the coupled particles. The intensity of the red channel (nanodiamond emission) increases by the number of coupled particles. The number of coupled particles can be controlled by the concentration of the target DNA during hybridization (inset). 0 T indicates 0 M concentration of the target DNA. (**c**) The ratio of the emission intensity of the nanodiamonds to the gold nanoparticles, defined as coupling efficiency, exhibits a non-linear dependence on the laser power, with a maximum at ~0.7 mW.

In the next step, we examined the optical coupling between the GNP–nanodiamond pairs under multiphoton illumination (Figure 3). The resonant LSP scattering of a GNP can radiate both into the far-field (detected by the microscope) and into the electronic states of the neighboring nanodiamonds (coupling). A strong modification of the luminescence spectra was observed in the coupled particles. A second peak at ~650 nm appeared, which matched well with the emission of the NV^−^ centers in the nanodiamonds. Consequently, the spectrum consisted of two peaks at ~520 and ~600–700 nm, which were correlated with the emission of the GNPs and the photoluminescence of nanodiamonds, respectively. The intensity of the nanodiamond emission increased by increasing the number of the coupled particles (i.e., by increasing the concentration of the target DNA in the hybridization assay: e.g., 0, 10^−15^, 10^−9^ and 10^−7^ M). For the non-assembled particles (i.e., 0 M concentration of the target DNA), the distance between the nanodiamond and the GNP was too large for an effective coupling, and thus the second peak did not appear. These results suggest that the excitation of the nanodiamonds under multiphoton irradiation was due to the near-field excitation by the neighboring GNPs.

Moreover, the coupling efficiency (I_nanodiamond_/I_GNP_) exhibited a nonlinear dependence on the laser power. By increasing the laser power, nanodiamond emission first increases (up to 70 μW laser power) and then decreases (while GNP emission increases with the power law). By plotting the relative intensity plots, a maximum point for the coupling efficiency could be found, suggesting a counteracting mechanism in the excitation/emission rates in nanodiamonds.

Further analyses are still necessary to characterize the coupled system, but the main question to answer would be “how the LSP resonances in GNP influence the emission in nanodiamonds?” The combination of the plasmonic resonances and the electronic states in a quantum emitter, such as a nanodiamond, is known to result in a multitude of coupling conditions. Depending on the distance between the components, photoluminescence intensity could be enhanced or quenched [45,46,47]. For example, previous studies have demonstrated that the emission lifetime of NV centers in nanodiamonds is reduced through a non-radiative decay mechanism via plasmonic coupling [30,31,32]. If the non-radiative energy transfer dominates, then the quantum yield (coupling efficiency) can be largely reduced. On the other hand, an increased excitation rate due to the enhanced local field can counteract the reduction in the quantum yield to some extent. 

The existence of competing rate regimes in Figure 3c can be qualitatively explained by examining the interplay between (i) the non-radiative decay rate and (ii) the excitation rate in plasmonically coupled nanodiamond emissions. To evaluate the role of LSPs in nanodiamond emission, two concurring effects have to be considered. On one hand, the excitation power in nanodiamonds is proportional to the emission power of the GNPs. On the other hand, both the radiative and non-radiative decay rates in nanodiamonds are modified by the local density of states (LDOS). Therefore, both emission power and LDOS are directly influenced by LSP resonances in GNPs.

## 5. Potential of the Coupled System for Nanoscopy

The instabilities in LSPs (which involve random switching between neutral and charged states) can lead to variations in the coupling strength and therefore the intensity and lifetime of the fluorescence in nanodiamonds. One could potentially exploit the emission instabilities to make the nanodiamonds blink. Indeed, it has been shown in other studies that (charge) fluctuations lead to fluorescence intermittency (e.g., random blinking in quantum dots) as a result of the dynamic radiative and non-radiative decay rates [48,49,50].

The major work associated with the use of NV centers for SRM was performed on bulk diamonds, and was mostly centered around stimulated emission depletion microscopy (STED) [51,52,53,54,55,56]. Using STED, subdiffraction resolution of 2.4 nm and 10 nmof NV centers were achieved in bulk diamond [57], and in nandiamonds, respectively. [56] Yet, STED microscopy is a destructive method for imaging biological samples due to the high required power of the lasers, on the order of 180 mW per frame [58].

Fluorescence intermittency in nanodiamonds can create new possibilities in stochastic optical reconstruction microscopy (STORM), which requires fast and bright blinking [59]. Fluorescence blinking occurs naturally in organic dyes due to photobleaching, and a photoswitching mechanism is enabled by means of UV-sensitive buffers (e.g., AlexaFluor647 switches between the “on” and “off” states ~26 times in mercaptoethylamine or MEA [60]). While the lifetime of the organic molecules is considered as a limiting factor in STORM, nanodiamonds can offer a robust platform for long term imaging, emitting ~10^5^ photons/s at room temperature with a relatively short excited electronic state lifetime (~10 ns) [12]. However, fluorescence blinking in nanodiamonds does not occur naturally at the time-scales and frequencies required for super-resolution microscopy. In a typical STORM experiment, hundreds of fluorophore blinking events occur over a ca. 30 msec acquisition time per frame.

There are currently two main approaches undertaken to induce blinking in nanodiamonds: (i) chemical modification of small nanodiamond particles (~5 nm in size) [61], and (ii) manipulating the electron spin state [62,63,64]. The former method has been shown to work for small nanodiamonds, where only a small fraction of them may host active optical defects. The latter approach mandates additional experimental setups for the optical detection of magnetic resonances (ODMR), and provides a moderate contrast of 18 % between the “on” and “off” states [62].

Gold nanoparticles, on the other hand, exhibit fast and strong flickering under multiphoton irradiation [40,65,66,67], which is attributed to the temporary damping of the plasmon resonance due to millisecond-length thermal fluctuations or the generation of “hot” electrons.

**Figure 4:**
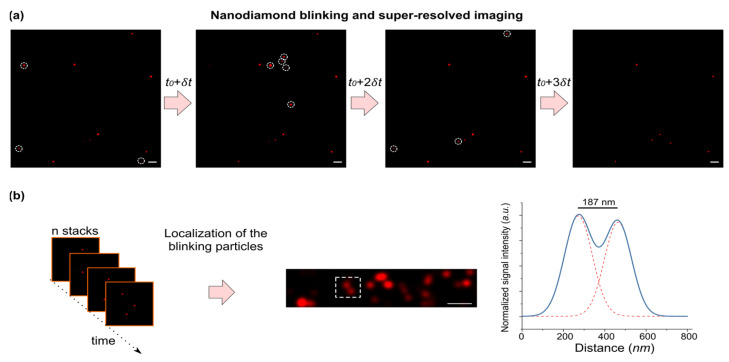
(**a**) A time-series of microscopic images in the nanodiamond channel. Some particles with fluctuating emissions are highlighted with a dashed circle. Scale bars, 10 µm. (**b**) (left) A localization algorithm can be applied on the image series for the detection of single particles. (middle) An example of a super-resolved image over time. (left) The intensity profile (of the region indicated in the middle image with dashed lines) is shown. Two fitted gaussian curves with a distance of 187 nm indicate the distance of two particles beyond the diffraction-limit (i.e., λ/2~325 nm).

One can therefore predict that, in a GNP–nanodiamond coupled system, photoluminescence in nanodiamonds is strongly influenced by the flickering of the coupled GNPs. Indeed, when we acquired images from the coupled systems at different time points, we noticed stochastic fluctuations in the intensity of emission in the nanodiamonds (Figure 4a). Blinking nanodiamonds could be utilized for STORM, by providing the possibility to switch between fluorescent and dark states. In a snapshot image, only a fraction of the nanodiamonds are detectable, whereas the rest of the nanodiamonds are in the dark state. As such, blinking is stochastic in nature, and one can acquire a stochastic map of emission by taking multiple snapshots. A time-series stack of images was used to analyze the temporal intensity of nanodiamond emissions. Image analysis was performed to localize the emission centers in each image via fitting, using the point spread function. As shown in the corresponding super-resolved image (Figure 4b), detection of multiple emitters beyond the diffraction limit is, in principle, plausible.

These fluctuations most likely originate from the fact that the electronic states in nanodiamond are strongly influenced by the temporal variations in the local surface plasmons discussed above. Although we are not able to characterize the emission fluctuations of nanodiamonds at this stage, it is envisioned that one can generate stochastic emission patterns via pulsed illuminations similar to the current state-of-the-art STORM setups [68], but with multiphoton lasers as the excitation source.

In summary, we introduced a new physical system in which gold nanoparticles and nanodiamonds formed conjugates via DNA hybridization. Harmonic mode emission generated in gold nanoparticles via a multiphoton process induced a coupled fluorescence emission in the nanodiamonds. Moreover, due to the instability of the harmonic modes in the gold nanoparticles, fluctuations in the emission can occur that would directly influence the nanodiamond emissions. A platform for super-resolution imaging is achievable via the utilization of stochastic emission fluctuations. As a proof of principle, a super-resolved image from the nanodiamonds was presented. It is envisioned that this platform could find utilization in intracellular biosensing and bioimaging applications (similar to other nanoparticle systems [69,70,71,72,73]), due to the DNA-based coupling mechanism and also the attractive characteristics of harmonic generation, such as low power, low background and tissue transparency.

## Data Availability

Not applicable.

## Supplementary Materials

The following supporting information can be downloaded at: https://www.mdpi.com/article/10.3390/bios12030148/s1, Table S1. Oligonucleotides used for the functionalization of GNPs and nanodiamonds as well as target strands for coupling the particles. SH denotes the thiol group on thiolated oligonucleotides. Colored bases match the target regions complementary to DNA1 and DNA2. Figure S1. The hybridization chambers consisting of PDMS microwells are shown schematically (sideview). Coverslips were placed on top of the filled chambers. After overnight incubation, the PDMS wells were removed and coverslips were replaced for microscopy imaging. Figure S2. Shows high-resolution transmission electron microscope (HR-TEM) images of: (a) aggregated nanodiamonds after drying on a TEM membrane; (b) crystal structure in a single nanodiamond; (c) a gold nanoparticle coupled to nanodiamonds. Gold nanoparticles have a high scattering cross section to the electrons (due to the high atomic number), therefore they appear dark in the transmission image. The nanodiamonds, on the other hand, are highly transparent to the electrons, creating a low contrast compared to the background. Figure S3. A dark-field image of gold nanoparticles under white-light illumination. The corresponding far-field spectrum of a single particle is shown on the right. Scale bar, 10 µm. Figure S4. Gold nanoparticles and nanodiamonds were illuminated with a multiphoton laser separately and before hybridization. GNPs show wavelength-dependent emission. Particularly, a strong signal is visible when the gold particles are illuminated with a wavelength in the range of 1000–1100 nm. It was found that illumination at 1020 nm produces the strongest signal. Nanodiamonds, on the other hand, do not show any detectable levels of illumination within the tested wavelength range. Figure S5. Multiple multipole program (MMP 6) simulation of the coupled GNP-nanodiamond system with one-photon illumination at different wavelengths. GNP (ε = 5.66) and nanodiamond (ε from Johnson&Christy7) are 50 and 15 nm in size, respectively. The gap between the particles is 5 nm. The incident angle and polarization of the light are shown with arrows. The intensity profiles of the Total Time Averaged Electric Field are shown (0–200 W/m^2^). The highest intensity profile corresponds to λexcitation= 520 nm, which is due to the excitation of the surface plasmons in a gold nanoparticle. Figure S6. Dynamical Light Scattering (DLS) measurements show the hydrodynamic diameter of nanodiamonds, GNPs and the coupled system [74,75,76].
