# Supplementary material for: Self-Assembly of Nanodiamonds and Plasmonic Nanoparticles for Nanoscopy
Source: Biosensors (Basel). 2022 Feb 28;12(3):148. doi: 10.3390/bios12030148 (PMC8946096; doi:10.3390/bios12030148)
Supplement: Supplementary file 1 [file biosensors-12-00148-s001.zip › biosensors-1594915-supplementary.pdf]

*Supplementary Materials*

# Self-Assembly of Nanodiamonds and Plasmonic Nanoparticles for Nanoscopy

**Lukas Schmidheini [1,†], Raphael F. Tiefenauer [1,†], Volker Gatterdam [1,†], Andreas Frutiger [1], Takumi Sannomiya [2] and Morteza Aramesh [1,3,*]**

[1] Laboratory of Biosensors and Bioelectronics, Institute for Biomedical Engineering, ETH Zürich, 8092 Zürich, Switzerland; lukas.schmidheini@biol.ethz.ch (L.S.); raphael.tiefenauer@gmail.com (R.F.T.); v.gatterdam@lino-biotech.com (V.G.); frutiger@biomed.ee.ethz.ch (A.F.)

[2] Department of Materials Science and Engineering, School of Materials and Chemical Technology, Tokyo Institute of Technology, Yokohama 226-8503, Japan; sannomiya.t.aa@m.titech.ac.jp

[3] Department of Materials Science and Engineering, Division of Biomedical Engineering, Uppsala University, 751 21 Uppsala, Sweden

* Correspondence: maramesh@ethz.ch

† These authors contributed equally to this work.

**Citation:** Schmidheini, L.; Tiefenauer, R.F.; Gatterdam, V.; Frutiger, A.; Sannomiya, T.; Aramesh, M. Self-Assembly of Nanodiamonds and Plasmonic Nanoparticles for Nanoscopy. *Biosensors* **2022**, *12*, 148. https://doi.org/10.3390/bios12030148

Received: 27 January 2022

Accepted: 24 February 2022

Published: 28 February 2022

**Publisher's Note:** MDPI stays neutral with regard to jurisdictional claims in published maps and institutional affiliations.

**Copyright:** © 2022 by the authors. Submitted for possible open access publication under the terms and conditions of the Creative Commons Attribution (CC BY) license (https://creativecommons.org/licenses/by/4.0/).

## 1. Experimental

***Materials and reagents:*** Thiolated oligonucleotide and target sequences were procured from Microsynth AG (Balgach, Switzerland). Gold nanoparticles with 49.2 nm mean diameter and ≤ 8% coefficient variation were purchased from BBI Solutions (Cardiff, UK). Tris(2-carboxyethyl)phosphine (TCEP), sodium dodecyl sulfate (SDS), glycerol, sodium chloride, phosphate buffered saline (PBS, from tabs), DNA LoBind tubes, Borane tetrahydrofuran (BH3·THF) complex solution (1.0 M) in THF, Sulfosuccinimidyl-4-(N-maleimidomethyl)cyclohexane-1-carboxylate (SSMCC), Triethanolamine (TEA) and dry acetone were procured from Sigma-Aldrich (Buchs, Switzerland). Cobas Integra Cleaner was procured from Roche Diagnostics (Basel, Switzerland). Polydimethylsiloxane (PDMS) was made from a Sylgard 184 silicone elastomer kit from Dow Corning (Midland, MI, USA). Powders of detonation nanodiamonds (grade G01) were procured from PlasmaChem GmbH (Berlin, Germany). All aqueous solutions were made with purified water (Milli-Q) from an Elix-5 water purification system. Prior to use, all buffer solutions were filtered with Millex-VV 0.1 μm filters from Faust Laborbedarf AG (Schaffhausen, Switzerland).

***DNA sequences:*** Table 1 contains the capture probe and analyte sequences used in this study, following the design of Storhoff et al.[1] DNA1 and DNA2 were 22 base-long oligonucleotides with 10 base-long poly-A spacer tails to increase the hybridization efficiency to the target region by increasing the accessibility. The target DNA consisted of 12 complementary bases to DNA1 and 12 complementary bases to DNA2. The ssDNA-SH was synthesized, HPLC-separated, reduced in DTT(dithiothreitol), NAP-5 separated, lyophilized, and sent under argon.

**Table S1.** Oligonucleotides used for the functionalization of GNPs and nanodiamonds, as well as target strands for coupling the particles. SH denotes the thiol group on thiolated oligonucleotides. Colored bases match the target regions complementary to DNA1 and DNA2.

| Name | | Sequence | |
|---|---|---|---|
| **DNA1** | **5'-** | TCTCAACTCGTAAAAAAAAAAA | -SH-3' |
| DNA2 | 5'-SH- | AAAAAAAAAACGCATTCAGGAT | -3' |
| Target DNA | 5'- | TACGAGTTGAGAATCCTGAATGCG | -3' |

***Gold nanoparticle functionalization:*** Thiolated DNA sequences (DNA2: 5′SH-A AAA CGC ATT CAG GAT-3′) were reduced in 2 mM TCEP prior to overnight incubation with 50 nm GNPs (4.5×1010 particles/ml) at 0.01% SDS. Subsequently, the solution was adjusted to 1 M NaCl in 10 mM PBS over 12 hours by salt aging, with a 10 s sonication step after each addition to ensure a high capture probe density. After a final wash step, the solution was restored to the initial concentration of the GNP solution in 1 M NaCl and 10 mM PBS at pH 7.4. The functionalized nanoparticles were stored in DNA LoBind tubes on a platform shaker at 100 rpm at room temperature.

***Nanodiamond functionalization:*** The surface of the as-received nanodiamonds were oxidized by annealing in air at 600 °C for 6 h.[2] The annealed powders were dispersed in Milli-Q water (resistance >18 Ω) at a concentration of 1 g/L. The solutions were ultrasonicated in Badelin SONOPLUS overnight. The suspension was centrifuged at 5000 rcf for 1 h and the supernatant was collected and lyophilized for further processing. Amine group functionalization was performed according to a process noted in the literature.[3] The lyophilized particles (1 g) were placed in a two-necked flask under a nitrogen flow. A solution of 1 M BH3·THF in THF (20 mL) was added dropwise to the particles. The solution was under reflux and stirring for 24 h. The mixture was cooled to room temperature and neutralized with 2 N hydrochloric acid. Hydrochloric acid was added until no hydrogen bubbles were observed. The mixture was centrifuged and washed with acetone and water (minimum 3 cycles), until the supernatant reached pH 7. The reduced nanodiamonds were then lyophilized. (3-aminopropyl)trimethoxysilane (10 mL, 10% solution in dry acetone) was added to 100 mg of the lyophilized particles and the solution was stirred under nitrogen flow for 48 h at room temperature. The samples were then washed with acetone three times and once with water. A light gray powder was achieved after lyophilization. DNA was attached to the amine-terminated nanodiamonds using a cross-linker.[4] A 14 nM Sulfosuccinimidyl-4-(N-maleimidomethyl)cyclohexane-1-carboxylate (SSMCC) was used as the cross-linker in a 0.1 M pH 7 triethanolamine (TEA) buffer. The nanodiamond concentration was adjusted to $4.5\times10^{10}$ particles/ml. The NHS-ester group of the cross-linker reacted specifically with the $-NH_2$ groups on the surface and formed amide bonds. The reaction was performed for 90 min at room temperature. The maleimide end reacted with thiol-modified DNA (DNA1) which was treated with TCEP, similar to the process for GNP functionalization with DNA2. The particle concentration was restored similar to that of the GNPs.

***Hybridization assay:*** Functionalized nanodiamonds (with DNA1) and GNP (with DNA2) were mixed (1 part each) with different concentrations of the target sequence (5′-TAC GAG TTG AGA ATC CTG AAT GCG-3′), followed by a heat cycle of 10 min at 75 °C and 4 hours at 25 °C (Biometra TPersonal Combi Thermocycler, Biometra GmbH, Göttingen, Germany). Thereafter, the mixture was directly used for analysis.

***Dynamic light scattering:*** A Malvern Zetasizer Nano ZS fitted with a 633 nm laser was used to determine the mean size and charge of the particles in the colloids by dynamic light scattering (DLS).

***Transmission electron microscopy:*** HRTEM analysis was carried out in a Hitachi (HD 2700) 200 kV system. TEM specimens were prepared by dipping TEM grids with holey carbon support film into the particle solution for 10 min.

***Sample preparation for microscopy:*** The microscopy slides and coverslips (Menzel-Gläser, Gerhard Menzel GmbH, Braunschweig, Germany) were cleaned with dust-free wipes using Cobas Integra Cleaner (1:10 in purified water) and ethanol followed by blow-drying with nitrogen. PDMS sheets (2-3 mm in height) were cut to size and wells were stamped out with a hole puncher (2.5 mm diameter). The PDMS wells were covered with coverslips after adding the sample solutions and left for overnight settling. On the following day, the solution and PDMS wells were removed, the coverslips were replaced, and the sample was used for microscopy.

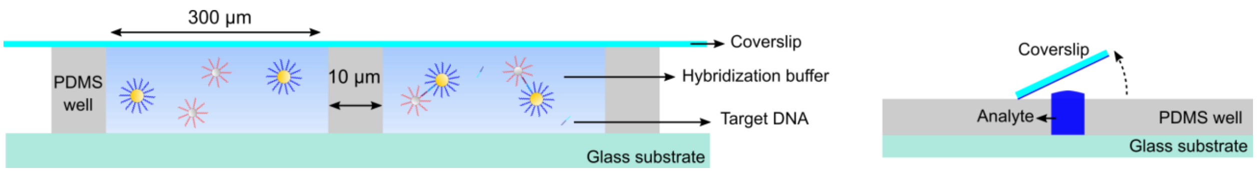


**Figure S1.** The hybridization chambers consisting of PDMS microwells are shown schematically (sideview). Coverslips were placed on top of the filled chambers. After overnight incubation, the PDMS wells were removed and the coverslips were replaced for microscopy imaging.

***Dark-field microscopy:*** A hyperspectral nanoscale optical imaging system consisting of an Olympus BX53F microscope, a dark-field optical illumination system (NA= 1.2 - 1.4, CytoViva Inc., Auburn, AL, USA), a 60x oil immersion objective (Olympus UPlanFLN 60x/1.25 Oil Iris), a halogen lamp (HAL 100), a CMOS camera and a Spectra-Pro Spectrophotometer (PIXIS 400, Princeton Instruments, USA) was used for image acquisition in dark-field mode.

***Multiphoton microscopy:*** Multiphoton microscopy was performed on a Leica TCS SP8 setup (Leica Microsystems GmBH, Wetzlar, Germany) that consisted of an upright microscope, two infrared pulsed lasers for multiphoton acquisitions, and sensitive Leica Hybrid detectors (HyD SP GaAsP). The laser beam was focused on the sample via an electro-optical modulator (EOM) to control the laser output and was coupled into the spectral scan-head passing through two galvoscanners (for the x–y plane). A Mai Tai XF (Spectra-Physics) was used for the 720–950 nm illumination range, and an InSight DeepSee (Spectra-Physics) was used for the 950–1300 nm range; both lasers were operated with a < 100-fs pulse duration and 80-MHz repetition rate. The illumination intensity was controlled by varying the laser power. A 40× 1.1 NA Water (HC PL IRAPO CORR) and a 63× 1.4 NA Oil (CS2 HC PL APO) objective (coverslip corrected) were used for imaging. To investigate the multiphoton absorption of the nanoparticles, the illumination wavelength was swept from 800–1300 nm. For the rest of the experiments, 1020 nm illumination was used, and the emitted signal from the samples were spectrally separated by a beam splitter (AOBS Acousto-Optic-Beam Splitter) before being detected by two Leica HyD detectors at different channels. Images were acquired using 100 Hz exposure time, and each data point was imaged with 3 independent samples and 3 images per sample.

The time-lapse images were obtained at 8000 Hz acquisition rates with a resonant scanner. Typically, an area of 35.23 × 6.2 $\mu m^2$ was scanned at 105 nm pixel size and a pixel dwell time of 110 nsec. Images were line-averaged 3 times to increase the signal-to-noise ratio. The total time interval was 15 msec per frame (~20,000 total frames).

***Image analysis:*** All the image analysis was performed using Fiji ImageJ open source software.[5] Thunderstorm was used for the generation of the point spread function and the localization of the emission centers.

**2. Supplementary Figures and Notes:**

*2.1. HR-TEM Images*

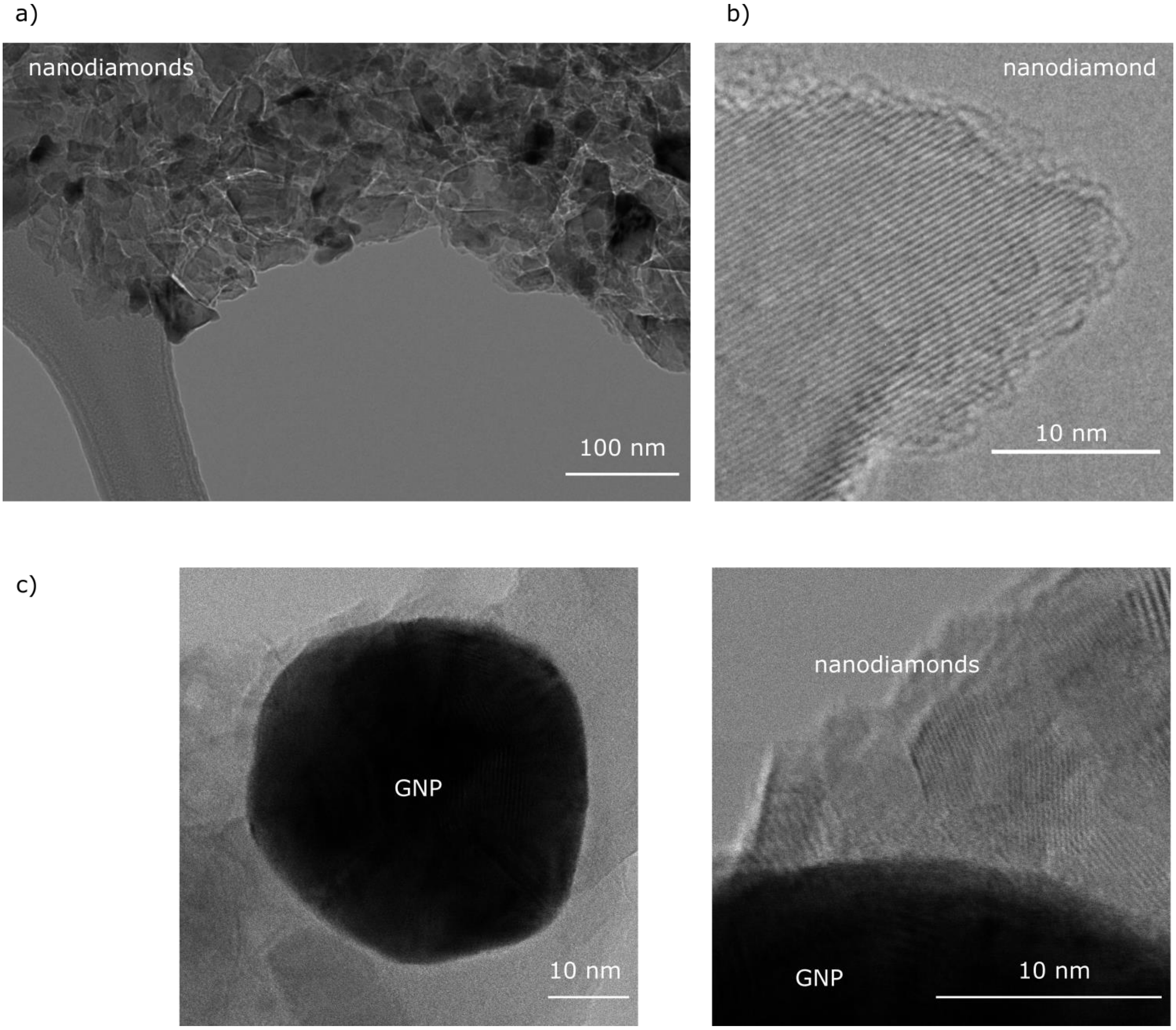


**Figure S2.** High-resolution transmission electron microscope (HR-TEM) images of: a) aggregated nanodiamonds after drying on a TEM membrane; b) crystal structure in a single nanodiamond; c) a gold nanoparticle coupled to nanodiamonds. Gold nanoparticles have a high scattering cross section to the electrons (due to the high atomic number), therefore they appear dark in the transmission image. The nanodiamonds, on the other hand, are highly transparent to the electrons, creating a low contrast compared to the background.

### 2.2. Dark-Field Images of GNPs

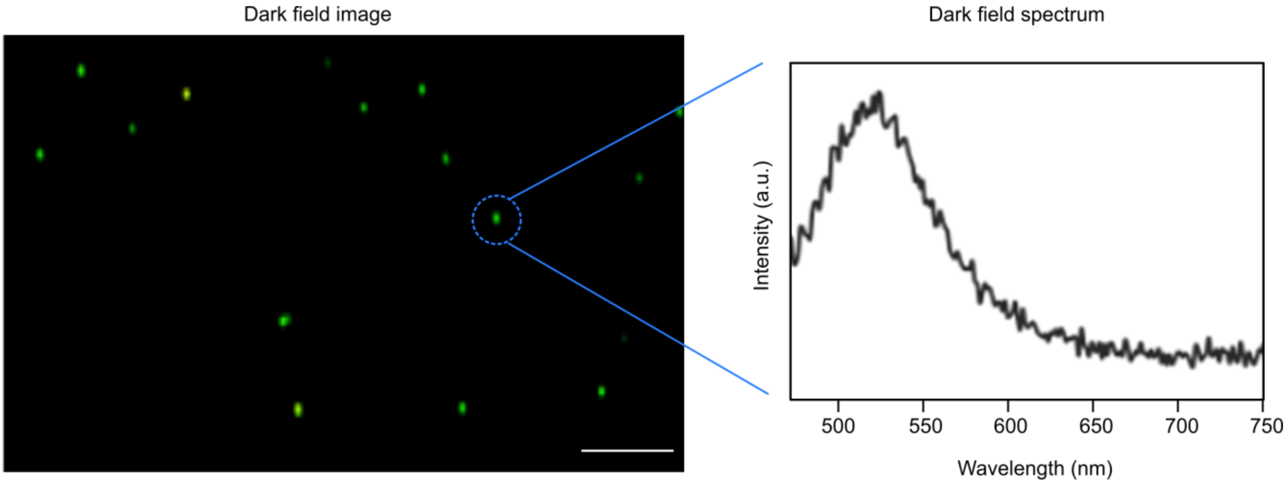


**Figure S3.** A dark-field image of gold nanoparticles under white-light illumination. The corresponding far-field spectrum of a single particle is shown on the right. Scale bar, 10 µm.

### 2.3. Wavelength Dependence of Second Harmonic Generation

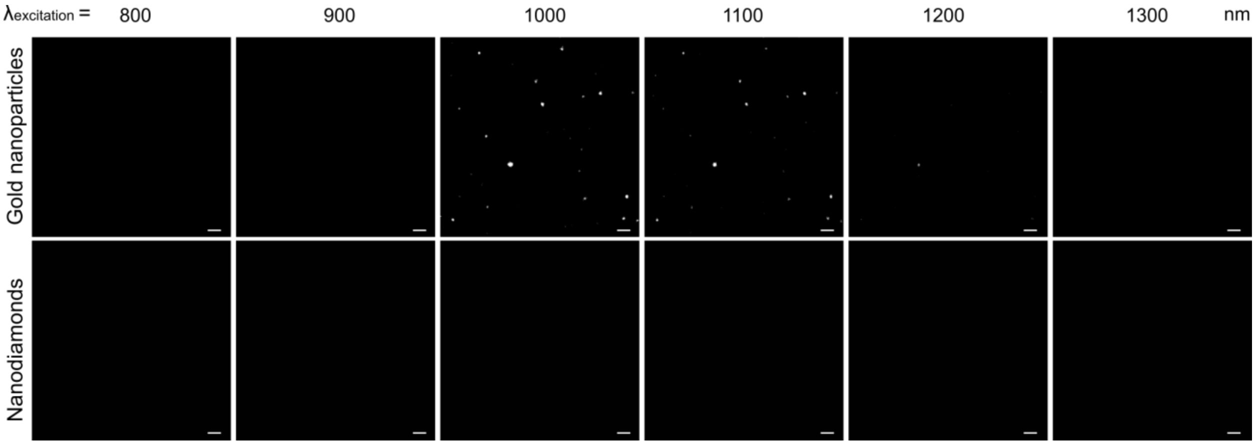


**Figure S4.** Gold nanoparticles and nanodiamonds were illuminated with a multiphoton laser separately and before hybridization. GNPs show wavelength-dependent emission. Particularly, a strong signal is visible when the gold particles are illuminated with a wavelength in the range of 1000–1100 nm. It was found that illumination at 1020 nm produced the strongest signal. Nanodiamonds, on the other hand, did not show any detectable levels of illumination within the tested wavelength range.

### *2.4. MMP Simulations*

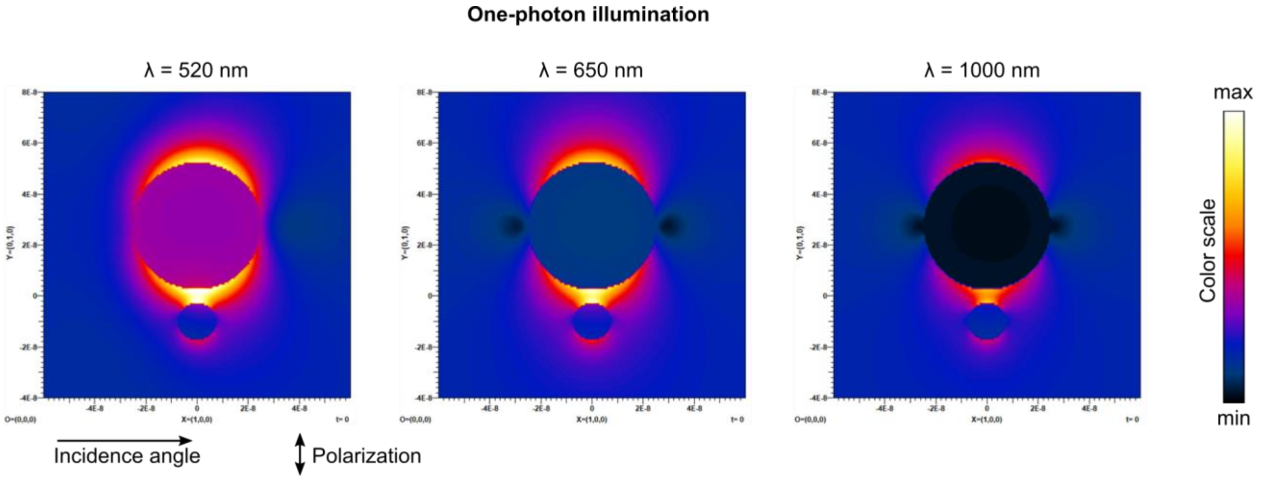


**Figure S5.** Multiple multipole program (MMP[6]) simulation of the coupled GNP-nanodiamond system with one-photon illumination at different wavelengths. The GNP ($\varepsilon$ = 5.66) and nanodiamond ($\varepsilon$ from Johnson&Christy[7]) samples were 50 and 15 nm in size, respectively. The gap between the particles was 5 nm. The incident angle and polarization of the light are shown with arrows. The intensity profiles of the Total Time Averaged Electric Field are shown (0-200 W/m$^2$). The highest intensity profile corresponded to $\lambda_{excitation}$= 520 nm, which was due to the excitation of the surface plasmons in a gold nanoparticle.

### *2.5. DLS Measurements*

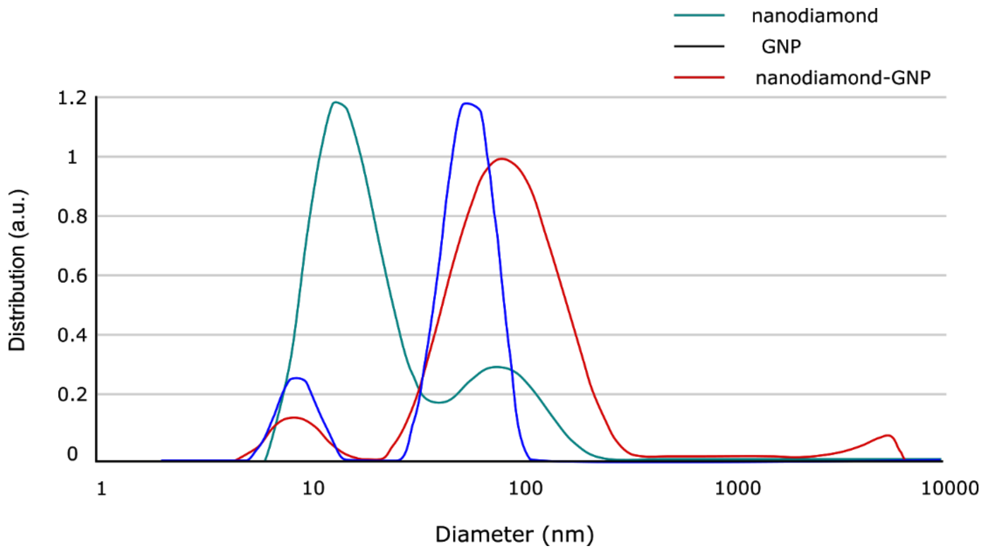


**Figure S6.** Dynamical Light Scattering (DLS) measurements show the hydrodynamic diameter of the nanodiamonds, the GNPs and the coupled system.

.

## 3. References


1. Storhoff, J. J., Elghanian, R., Mucic, R. C., Mirkin, C. A. & Letsinger, R. L. One-Pot Colorimetric Differentiation of Polynucleotides with Single Base Imperfections Using Gold Nanoparticle Probes. *J.* Vol. 120 1959–1964 (1998).
2. Aramesh, M., Shimoni, O., Ostrikov, K., Prawer, S. & Cervenka, J. Surface charge effects in protein adsorption on nanodiamonds. *Nanoscale* **7**, 5726–5736 (2015).
3. Zhang, X.-Q. *et al.* Polymer-functionalized nanodiamond platforms as vehicles for gene delivery. *ACS Nano* **3**, 2609–2616 (2009).
4. Knickerbocker, T. *et al.* DNA-Modified Diamond Surfaces. *Langmuir* vol. 19 1938–1942 (2003).
5. Rueden, C. T. *et al.* ImageJ2: ImageJ for the next generation of scientific image data. *BMC Bioinformatics* **18**, 529 (2017).
6. Sannomiya, T. & Hafner, C. Multiple Multipole Program Modelling for Nano Plasmonic Sensors. *J. Comput. Theor. Nanosci.* **7**, 1587–1595 (2010).
7. Johnson, P. B. & Christy, R. W. Optical Constants of the Noble Metals. *Physical Review B* vol. 6 4370–4379 (1972).
